# Lactic acid bacterial inoculant effects on the vitamin content of alfalfa and Chinese leymus silage

**DOI:** 10.5713/ajas.19.0135

**Published:** 2019-05-27

**Authors:** Tingting Jia, Zhiqiang Sun, Run Gao, Zhu Yu

**Affiliations:** 1 Department of grassland production and utilization, College of Grassland Science and Technology, China Agricultural University, Beijing 100193, China

**Keywords:** Alfalfa Silage, Chinese Leymus Silage, *Lactobacillus* Inoculants, Vitamin

## Abstract

**Objective:**

Information regarding the vitamin content of silage is limited. This study investigated the changes in the vitamin content of alfalfa and Chinese leymus silages with or without a lactic acid bacterial inoculant.

**Methods:**

Alfalfa at the early flowering stage and Chinese leymus at the full-bloom stage were harvested. The treatments for each forage type were control (deionized water only) and 1×10^6^ colony-forming units *Lactobacillus plantarum* (LP)/g fresh matter. After 45 days of ensiling, all silages were sampled for evaluating the vitamin content, fermentation quality and chemical composition.

**Results:**

The LP inoculant decreased the pH value and ammonia nitrogen content of the alfalfa and Chinese leymus silages and significantly (p<0.05) increased the lactic acid, acetic acid concentrations and Flieg’s points. Prior to ensiling, the levels of five B-group vitamins (thiamin, riboflavin, niacin, pantothenic acid, and pyridoxine) and α-tocopherol in alfalfa were significantly (p<0.01) higher than those in Chinese leymus. Ensiling decreased the levels of the five B-group vitamins in both alfalfa and Chinese leymus while increasing the α-tocopherol content of Chinese leymus. The thiamin, riboflavin, niacin and pantothenic acid levels in the LP-treated silage were significantly (p<0.05) lower than those in the untreated silage for the alfalfa and Chinese leymus. The α-tocopherol content in the LP-treated alfalfa silage was significantly (p<0.05) higher than that in the untreated alfalfa silage. There was no significant (p>0.05) difference in pyridoxine content between the untreated and LP-treated silages for both forages.

**Conclusion:**

With or without LP inoculation, the levels of the five B-group vitamins (thiamin, riboflavin, niacin, pantothenic acid, and pyridoxine) in alfalfa and Chinese leymus decreased after 45 days of ensiling, while the α-tocopherol content of Chinese leymus increased. The LP inoculant improved the fermentation quality of both the alfalfa and Chinese leymus silages but increased the thiamin, riboflavin, niacin, and pantothenic acid loss in the two forages after fermentation.

## INTRODUCTION

Vitamins are organic nutrients that are essential in minute quantities for the nutrition of ruminants [1]. Vitamins act as coenzymes and precursors of coenzymes in the regulation of metabolic processes [2]. Fresh or conserved forages are important dietary sources of natural vitamins in ruminants [1,3]. The concentrate used in ruminant diets does not contain any natural vitamins or vitamin precursors [4]. α-Tocopherol and β-carotene in forage have received much attention due to their antioxidant properties [3,5–9]. The level of α-tocopherol in ensiled forage was seen to be higher than that in hay [6,7]. There is little information regarding the levels of B-group vitamins in silage and the effects of additives on the B vitamin content. B-group vitamins are essential for ruminants, and vitamin B deficiency can lead to different degrees of metabolic disorders [7]. It has been shown that there is an increasing need for B vitamins in ruminants due to the increased demand for high productivity and quality of animal products [4]. However, to the best of our knowledge, there have been few studies on B-group vitamins in silage. Additional information is required regarding the changes in B vitamin levels in silage.

Alfalfa and Chinese leymus are the main forage sources for animal diets in China. Storage of this forage as silage is a good way to retain the nutritional content. Additives are added during forage ensilage to achieve high-quality silage. The additives strongly influence the fermentation process of silage and may influence the vitamin content in silage [7]. Shingfield et al [8] demonstrated an increase in the α-tocopherol content in silage via the use of an inoculant enzyme preparation. Liu et al [9] reported that the use of additives decreased the α-tocopherol and β-carotene levels in silages. However, there are no reports in the literature on the effect of lactic acid bacterial additives on B-group vitamins in silage. Certel et al [10] and Ochanda et al [11] found that fermentation influences the concentrations of B-group vitamins in food. However, information regarding B-group vitamins in silage is limited. Therefore, this study was undertaken to provide information on the effects of lactic acid bacterial inoculants on the vitamin content of alfalfa and Chinese leymus silage. Additionally, the fermentation quality and chemical composition of alfalfa and Chinese leymus silage were also evaluated.

## MATERIALS AND METHODS

### Silage materials and ensiling

Alfalfa and Chinese leymus were grown in Guyuan County (41°42′-41°57′N, 115°32′-115°59′E), Hebei Province, P. R. China. Alfalfa at the early flowering stage and Chinese leymus at the full-bloom stage were harvested with 4 to 5-cm stubble height and wilted for 2 hours. Then, the alfalfa and Chinese leymus forages were chopped into 1 to 2-cm pieces with a forage cutter. The treatments for each forage type were control (deionized water only) and *Lactobacillus plantarum* inoculant (LP). The lactic acid bacterial strain LP was isolated from the grass and identified as *L. plantarum* via 16S rDNA sequencing. The LP strain was inoculated at 1×10^6^ colony-forming units/g fresh matter. Approximately 300 g of chopped alfalfa and Chinese leymus forages were filled into polyethylene bag silos (30×40 cm; 0.19 mm thickness; 50 cm^3^ m^−2^ day^−1^ 0.1 Mpa^−1^). Each treatment had three repetitions. The silos were sealed with a vacuum sealer (FW3150; Fresh World Electric Co., Ltd., Guangzhou, China). All the silos were preserved at ambient temperature in a dark room, then opened and sampled after ensiling for 45 days.

### Chemical analysis

Twenty-gram samples of alfalfa and Chinese leymus silages were mixed with 180 mL of distilled water and then homogenized in a juicer for 2 min. The mixture was filtered, and then, the filtrate was used for determination of pH and organic acid and ammonia nitrogen concentrations. The pH was measured by using a pH meter (PHS-3C, INESA Scientific Instrument Co., Ltd., Shanghai, China). The organic acid (lactic, acetic, propionic and butyric acids) levels were determined by high performance liquid chromatography (HPLC; column: Shodex RS Pak KC-811, Showa Denko KK, Kawasaki, Japan; detector: DAD, 210 nm, SPD-20A, Shimadzu Co., Ltd., Kyoto, Japan; eluent: 3 mmol/L HClO_4_, at a flow rate of 1.0 mL/min; column temperature: 50°C). The ammonia nitrogen concentration was analyzed by the phenol and sodium hypochlorite method [12]. The buffering capacity of the alfalfa and Chinese leymus materials was determined by the method described by Playne and McDonald [13]. The dry matter (DM) content was measured after oven-drying at 65°C for 48 hours. The oven-dried samples were first milled and passed through a 1.0-mm screen and then used for analysis of the water-soluble carbohydrate (WSC), crude protein (CP), neutral detergent fiber (NDF), acid detergent fiber (ADF), and hemicellulose (HC) levels. The WSC content was measured by the anthrone method [14]. The CP content was analyzed by using method 976.05 of the Association of Official Analytical Chemists [15]. The NDF and ADF levels were measured by the method described by Van Soest et al [16]. The HC content of the samples was estimated as the NDF value minus the ADF value.

### Vitamin analysis

The vitamin concentrations were determined by HPLC. The sample (100 g) was freeze dried using a vacuum freeze-drying machine for 2 days (FreeZone 4.5L, LABCONCO Corp., Kansas City, MO, USA) before analysis. The freeze-dried samples were then ground and passed through a 1.0-mm screen.

Thiamin concentrations were determined in duplicate based on the method of GB/T 14700-2018. The extraction solution was prepared as follows: 107 g of NH_4_Cl was dissolved in 1,000 mL of ultrapure water; then, the pH was adjusted to 3 to 4 with 2 mol/L HCl; 900 mL of this NH_4_Cl solution was mixed with 100 mL of carbinol (Sigma-Aldrich, Darmstadt, Germany). Then, 5 g of sample and 70 mL of extraction solution were mixed in a 100-mL brown-glass volumetric flask. Next, the samples were incubated in an ultrasonic bath (30 min). After cooling in an ice bath, the volume was made up to 100 mL, and the mixture was centrifuged (8,000 r/min, 5 min). The supernatant was collected and passed through a 0.45-μm syringe filter. Twenty microliters of the filtered supernatant were injected into an HPLC instrument. The samples were examined on a Pursuit 5-μm C18 column (150×4.6 mm; Agilent, Ammerbuch, Germany). The mobile phase used was prepared as follows: 1.1 g of sodium heptanesulfonate and 50 mg of disodium ethylenediamine tetraacetic acid were dissolved in 700 mL of ultrapure water, and after total dissolution, 25 mL of glacial acetic acid and 10 mL of triethylamine (Sigma-Aldrich, Germany) were added. Then, the solution was made up to 1,000 mL, and the pH value was adjusted to 3.7. Then, 800 mL of this solution was mixed with 200 mL of carbinol (Sigma-Aldrich, Germany). The flow rate was 1.0 mL/min, and the diode array detector was operated at a wavelength of 242 nm. The column temperature was 25°C to 28°C.

Riboflavin levels were analyzed in duplicate according to the method of GB/T 14701-2002. The extraction solution was prepared as follows: 50 mg of disodium ethylenediamine tetraacetic acid was dissolved in 700 mL of ultrapure water, and after total dissolution, 25 mL of glacial acetic acid and 5 mL of triethylamine were added, and the volume was adjusted to 1,000 mL. Then, 5 g of sample and 30 mL of extraction solution were mixed in a 100-mL brown-glass volumetric flask. Next, the samples were incubated in an 80°C to 100°C water bath (30 min). After cooling in an ice bath, 14 mL of carbinol was added. Then, the volume was made up to 100 mL with extraction solution, and the solution was centrifuged (8,000 r/min, 5 min). The supernatant was collected and passed through a 0.45-μm syringe filter. Then, 10 μL of the filtered supernatant was injected into an HPLC instrument. The samples were examined on a Pursuit 5-μm C18 column (150×4.6 mm; Agilent, Germany). The mobile phase was prepared as follows: 1.1 g of sodium heptanesulfonate and 50 mg of disodium ethylenediamine tetraacetic acid was dissolved in 700 mL of ultrapure water, and after total dissolution, 25 mL of glacial acetic acid and 5 mL of triethylamine (Sigma-Aldrich, Germany) were added. The solution was then made up to 1,000 mL, and the pH value was adjusted to 3.4; 860 mL of this solution were mixed with 140 mL of carbinol (Sigma-Aldrich, Germany). The flow rate was 0.8 mL/min, and the diode array detector was operated at a wavelength of 280 nm. The column temperature was 25°C to 28°C.

Niacin levels were examined according to the method of GB/T 17813-2018. The extraction solution was prepared as follows: 50 mg of disodium ethylenediamine tetraacetic acid was dissolved in 800 mL of ultrapure water, and then, 20 mL of glacial acetic acid and 5 mL of triethylamine were added; after thorough mixing, this solution was mixed with 200 mL of carbinol (Sigma-Aldrich, Germany). Then, 5 g of sample, 1 g of disodium ethylenediamine tetraacetic acid and 70 mL of extraction solution were mixed in a 100-mL brown-glass volumetric flask. Next, the samples were incubated in an ultrasonic bath (15 min). After cooling in an ice bath, the volume was made up to 100 mL with extraction solution, and the mixture was centrifuged (8,000 r/min, 5 min). The supernatant was collected and passed through a 0.45-μm syringe filter. Then, 20 μL of the filtered supernatant was injected into an HPLC instrument. The samples were examined on a Pursuit 5-μm C18 column (150×4.6 mm; Agilent, Germany). The mobile phase was prepared as follows: 1.1 g of sodium heptanesulfonate and 50 mg of disodium ethylenediamine tetraacetic acid were dissolved in 1,000 mL of ultrapure water, and after total dissolution, 20 mL of glacial acetic acid and 5 mL of triethylamine (Sigma-Aldrich, Germany) were added, and the pH value was adjusted to 4.0. Then, 800 mL of this solution was mixed with 200 mL of carbinol (Sigma-Aldrich, Germany). The flow rate was 1.0 mL/min, and the diode array detector was operated at a wavelength of 262 nm.

Pantothenic acid levels were measured based on the method of GB/T 18397-2014. Five grams of sample and 50 mL of the mobile phase were mixed in a 150-mL conical flask. The samples were incubated in an ultrasonic bath (15 min). After cooling in an ice bath, the samples were centrifuged (8,000 r/min, 5 min). The supernatant was collected and passed through a 0.45-μm syringe filter. Then, 10 μL of the filtered supernatant was injected into an HPLC instrument. The samples were examined on a Pursuit 5-μm C18 column (150×4.6 mm; Agilent, Germany). The mobile phase was prepared by adding 50 mL of acetonitrile to 950 mL of 0.05% phosphoric acid (aqueous solution). The flow rate was 1.0 mL/min, and the diode array detector was operated at a wavelength of 200 nm.

Pyridoxine levels were determined based on the method of GB/T 14702-2002. The extraction solution was prepared as follows: 50 mg of disodium ethylenediamine tetraacetic acid was dissolved in 700 mL of ultrapure water, and then, 25 mL of glacial acetic acid and 5 mL of triethylamine were added, and the volume was adjusted to 1,000 mL. Then, 800 mL of this solution was mixed with 200 mL of carbinol (Sigma-Aldrich, Germany). Then, 5 g of sample and 70 mL of sodium dihydrogen phosphate solution were mixed in a 100-mL brown-glass volumetric flask. Next, the samples were incubated in an ultrasonic bath (20 min). After cooling in an ice bath, the volume was made up to 100 mL with extraction solution, and the mixture was centrifuged (8,000 r/min, 5 min). The supernatant was collected and passed through a 0.45-μm syringe filter. Then, 20 μL of filtered supernatant was injected into an HPLC instrument. The samples were examined on a Pursuit 5-μm C18 column (250×4.6 mm; Agilent, Germany). The mobile phase was prepared as follows: 1.1 g of sodium heptanesulfonate and 50 mg of disodium ethylenediamine tetraacetic acid were dissolved in 700 mL of ultrapure water, and after total dissolution, 25 mL of glacial acetic acid and 5 mL of triethylamine (Sigma-Aldrich, Germany) were added, and the volume was adjusted to 1,000 mL with ultrapure water. The pH was adjusted to 4.0. Then, 800 mL of this solution was mixed with 200 mL of carbinol (Sigma-Aldrich, Germany). The flow rate was 1.0 mL/min, and the diode array detector was operated at a wavelength of 290 nm.

The α-tocopherol levels were analyzed based on the method of GB/T 17812-2008. The extraction solution was prepared as follows: 50 mg of disodium ethylenediamine tetraacetic acid was dissolved in 700 mL of ultrapure water, and then, 25 mL of glacial acetic acid and 5 mL of triethylamine were added, and the volume was adjusted to 1,000 mL. Then, 800 mL of this solution was mixed with 200 mL of carbinol (Sigma-Aldrich, Germany). Then, 5 g of sample and 80 mL of carbinol were mixed in a 100-mL brown-glass volumetric flask. Next, the samples were incubated in an ultrasonic bath (60°C, 20 min). After cooling in an ice bath, the volume was made up to 100 mL with carbinol, and the solution was centrifuged (8,000 r/min, 5 min). The supernatant was collected and passed through a 0.45-μm syringe filter. Then, 20 μL of the filtered supernatant was injected into and HPLC instrument. The samples were examined on a Pursuit 5-μm C18 column (250×4.6 mm; Agilent, Germany). The mobile phase was composed of 98 mL of carbinol and 2 mL of ultrapure water. The flow rate was 1.0 mL/min, and the diode array detector was operated at a wavelength of 285 nm.

### Calculation of the Flieg’s point

The quality of the alfalfa and Chinese leymus silage was estimated based on the Flieg’s point index, which was calculated by using the following equation [17]:

$$\text{Flie}{\text{g}}^{’}\text{s point}=220+[(2\times \%\;\text{DM})-15]-40\times \text{pH}$$

### Statistical analysis

The vitamin content, fermentation quality, and chemical composition data were analyzed by analysis of variance (ANOVA) using the general linear model-univariate procedure of SPSS 19.0 software [18]. ANOVAs were performed for forage types and treatments (control vs LP inoculant) as the two main parameters and for the interaction between the two parameters. The mean values were compared using Duncan’s multiple-range tests. Differences between means were considered significant when p<0.05.

## RESULTS

### Chemical composition and vitamin content of alfalfa and Chinese leymus forages before ensiling

The chemical composition and vitamin content of the alfalfa and Chinese leymus forages before ensiling are summarized in Table 1. The DM levels in the alfalfa and Chinese leymus were 358.2 and 450.1 g/kg, respectively. The buffering capacity of alfalfa was significantly (p<0.001) higher than that of Chinese leymus. The NDF, ADF, HC, and WSC levels in alfalfa were significantly (p<0.01) lower than those in Chinese leymus. The CP level in alfalfa was significantly (p<0.001) higher than that in Chinese leymus. The thiamin, riboflavin, niacin, pantothenic acid, pyridoxine and α-tocopherol concentrations in alfalfa were significantly (p<0.01) higher than those in Chinese leymus.

### Fermentation characteristics of alfalfa and Chinese leymus silages

The fermentation characteristics of the alfalfa and Chinese leymus silages are presented in Table 2. The LP inoculant significantly (p<0.05) decreased the pH values and significantly (p<0.05) increased the lactic acid and acetic acid levels of the alfalfa and Chinese leymus silages. For the Chinese leymus silage, the butyric acid levels and ratio of lactic acid levels to acetic acid levels in the LP-treated silage were significantly (p<0.05) lower those in the control. For the alfalfa silage, the ammonia nitrogen content was significantly (p<0.01) lowered by the LP inoculant. The pH value of the Chinese leymus silage was significantly (p<0.05) lower than that of the alfalfa silage, and the lactic acid, acetic acid and ammonia nitrogen levels in the Chinese leymus silage were significantly (p<0.05) lower than those in the alfalfa silage. The LP inoculants significantly (p<0.05) increased the Flieg’s points of the alfalfa and Chinese silages, and the Flieg’s point of the Chinese leymus silage was significantly (p<0.001) higher than that of the alfalfa silage.

### Chemical compositions of alfalfa and Chinese leymus silages

The chemical compositions of the alfalfa and Chinese leymus silages are shown in Table 3. There was no significant difference (p>0.05) in DM, WSC, NDF, and HC levels between the control and LP treatments of the alfalfa and Chinese leymus silages. For the alfalfa silage, the CP content of the LP treatment was significantly (p<0.05) higher than that of the control. For the Chinese leymus silage, the ADF content of the LP treatment was significantly (p<0.01) lower than that of the control.

### Vitamin concentrations of alfalfa and Chinese leymus silages

The vitamin concentrations in the alfalfa and Chinese leymus silages are listed in Table 4. After 45 days of ensiling, the thiamin, riboflavin, niacin, pantothenic acid and pyridoxine concentrations in the alfalfa and Chinese leymus silages were significantly (p<0.05) lower than those in the corresponding raw materials. Upon ensilage without the LP inoculant, the thiamin, riboflavin, niacin, pantothenic acid, pyridoxine and α-tocopherol levels in alfalfa decreased by 72.1%, 42.2%, 61.9%, 42.2%, 57.6%, and 30.7%, respectively, after 45 days of fermentation; upon inoculation with LP, the thiamin, riboflavin, niacin, pantothenic acid, pyridoxine, and α-tocopherol levels decreased by 81.2%, 52.9%, 71.4%, 53.2%, 58.8%, and 8.0%, respectively. Upon ensilage without the LP inoculant, the thiamin, riboflavin, niacin, pantothenic acid, and pyridoxine levels in Chinese leymus decreased by 54.1%, 60.2%, 64.8%, 60.3%, and 66.4%, respectively, after 45 days of fermentation, while inoculation with LP decreased the thiamin, riboflavin, niacin, pantothenic acid, and pyridoxine levels by 59.4%, 65.1%, 90.4%, 66.4%, 65.2%, respectively. However, the α-tocopherol content of untreated and LP-treated Chinese leymus silage increased by 52.1% and 49.9%, respectively. After 45 days of ensiling, the thiamin, riboflavin, niacin and pantothenic acid content of LP-treated silage was significantly (p<0.05) lower than that of untreated silage for both alfalfa and Chinese leymus silages. The α-tocopherol content of LP-treated silage was significantly (p<0.05) higher than that of untreated silage for the alfalfa silage. There was no significant (p>0.05) difference in pyridoxine content between the control and LP treatments for the alfalfa and Chinese leymus silages.

## DISCUSSION

By the end of ensiling, the addition of LP inoculant had a positive effect on alfalfa and Chinese leymus silage fermentation, as demonstrated by the low pH and butyric acid and ammonia nitrogen content and high lactic acid concentration in the inoculated silage. After 45 days of ensiling, the loss of WSC content in both forages was approximately 80%, which was due to the conversion of WSC to organic acid, while there was no significant difference in residual WSC content between the control and LP-treated silages. The pH of the alfalfa silage was much higher than that of the Chinese leymus silage, probably due to the high buffering capacity and low WSC content of alfalfa. The butyric acid and ammonia nitrogen concentrations in the alfalfa silage were significantly higher than those in the Chinese leymus silage in the present study, which indicates that alfalfa undergoes a greater degree of proteolysis than Chinese leymus. This result is consistent with previous observations of Papadopoulos and Mckersie [19] and can perhaps be explained by the high susceptibility of alfalfa proteins to proteolysis [20]. The higher acetic acid content and lower ratio of lactic acid to acetic acid in the control alfalfa silage than in the control Chinese leymus silage were indicative of the activity of heterofermentative lactic acid bacteria. Based on the Flieg’s points of the alfalfa and Chinese leymus silages, the fermentation quality of the Chinese leymus silage was better than that of the alfalfa silage, and the use of LP inoculant could improve the fermentation quality of both silages.

Many studies have investigated the α-tocopherol and β-carotene present in silage [6–9], while the B vitamins in alfalfa and Chinese leymus silages have not yet been discussed in the literature [7]. The thiamine concentrations in the alfalfa and Chinese leymus silages were 2.475 and 1.663 mg/kg DM, respectively, which were close to the level (1.37 mg/kg DM) reported by Beaudet et al [21]. The riboflavin levels in the alfalfa and Chinese leymus silages were 52.21 and 42.53 mg/kg DM, respectively, which were higher than the value reported by Schwab et al [22] in corn silage (3.5 mg/kg DM) but similar to the value reported by Beaudet et al [21] in corn silage (73.2 mg/kg DM). The niacin levels in the alfalfa and Chinese leymus silages were 0.743 and 0.558 mg/kg DM, respectively, which were lower than value (1.1 to 34 mg/kg) previously reported by Ballet al [7] in silage. The pantothenic acid levels in the alfalfa and Chinese leymus silages were 18.38 and 14.89 mg/kg DM, respectively, which were higher than the value reported by Schwab et al [22] in corn silage (1.5 mg/kg DM). The pyridoxine levels in the alfalfa and Chinese leymus silages were 1.140 and 1.108 mg/kg DM, respectively, which were lower than the value reported by Schwab et al [22] in corn silage (1.9 mg/kg DM). Based on the results of the present study, the thiamin, riboflavin, niacin, pantothenic acid, pyridoxine and α-tocopherol levels in the alfalfa forage were significantly higher than those in the Chinese leymus. The observed variability in B vitamin levels in silage suggests that many factors contribute to the variability, including forage species, climatic conditions, maturity and storage methods [7]. After 45 days of ensiling, the thiamin, riboflavin, niacin, pantothenic acid and pyridoxine concentrations in the untreated alfalfa silage decreased by 72.1%, 42.2%, 61.9%, 42.2%, and 57.6%, respectively. For the untreated Chinese leymus silage, the levels of thiamin, riboflavin, niacin, pantothenic acid and pyridoxine decreased by 54.1%, 60.2%, 64.8%, 60.3%, and 66.4%, respectively. Except for pyridoxine in Chinese leymus, the loss of B vitamins increased upon inoculation with LP. The decrease in thiamine content in the present study was similar to the results of Rao and Basu [23], who found that thiamine levels decreased during fermentation with lactobacilli. However, the reason underlying the observed influence of the lactic acid bacterial inoculant on B vitamin content in the present study. The continued increase in dairy productivity and quality of ruminants has resulted in increased B vitamin requirements [7]. Many studies conducted on other fermentation products, such as yogurt, cheeses, tarhana, and cereals [10,24–26], have shown that the levels of B-group vitamins in these fermented products increased after fermentation due to the synthesis of B vitamins by selected lactic acid bacteria [26]. Therefore, it may be possible to select B-vitamin-producing lactic acid bacteria to increase the B vitamin concentrations in silage.

α-Tocopherol is an important antioxidant. The α-tocopherol content of the Chinese leymus forage was similar to the value reported by Ballet et al [7] in grasses harvested during the early to late flowering stages but higher than the value reported by Liu et al [9] for napier grass. After 45 days of fermentation, the α-tocopherol content of the untreated Chinese leymus silage increased by 52.1% compared with that of raw Chinese leymus. To date, there has been no clear explanation for the changes in α-tocopherol levels observed during ensiling. Lindqvist et al [27] hypothesized that α-tocopherol-producing microorganisms exist on plants [28]. However, after 45 days of fermentation, the α-tocopherol content of the untreated alfalfa silage decreased by 30.7% compared with that of raw alfalfa, while the α-tocopherol content of the LP-treated alfalfa silage decreased by 8.0%; perhaps the α-tocopherol content is associated with the pH of silage. Liu et al [9] found that the residual rate of α-tocopherol was high at pH 4.0, and higher or lower pH values decreased the residual rate of α-tocopherol. Therefore, the high pH of LP-treated and untreated alfalfa silage (4.82 and 5.22, respectively) resulted in loss of α-tocopherol in alfalfa silage. The LP-treated silage retained a higher amount of α-tocopherol than the control silage due to the low pH of the LP-treated silage.

In conclusion, prior to ensiling, the levels of five B-group vitamins (thiamin, riboflavin, niacin, pantothenic acid, and pyridoxine) and α-tocopherol in alfalfa were significantly higher than those in Chinese leymus. With or without LP inoculation, the levels of the five B-group vitamins in alfalfa and Chinese leymus decreased after 45 days of ensiling, while the α-tocopherol content of Chinese leymus increased. The LP inoculant improved the fermentation quality of both the alfalfa and Chinese leymus silages but increased the thiamin, riboflavin, niacin and pantothenic acid loss in the two forages after 45 days of fermentation. In addition, after fermentation, the α-tocopherol content of alfalfa decreased, but the LP-treated silage retained more α-tocopherol than the untreated silage.

## Figures and Tables

**Table 1:** Chemical composition and vitamin content of alfalfa and Chinese leymus prior to treatment and ensiling

Items	Forage		SEM	p-value

	Alfalfa	Chinese leymus		
Dry matter (g/kg)	358.2±5.65	450.1±0.71	5.70	<0.001
Buffering capacity (mEq/kg DM)	947.4±73.2	296.0±6.70	73.5	<0.001
Chemical composition (g/kg DM)				
Neutral detergent fiber	374.2±4.39	630.8±8.02	9.14	0.001
Acid detergent fiber	269.9±1.52	324.3±3.94	3.53	<0.001
Hemicellulose	105.6±5.76	296.6±1.97	6.09	0.001
Crude protein	229.0±0.73	67.1±0.34	0.81	<0.001
Water-soluble carbohydrates	37.30±1.07	81.3±0.02	1.07	<0.001
Vitamins content (mg/kg DM)				
Thiamin	8.858±0.28	5.317±0.19	0.33	<0.001
Riboflavin	90.35±1.10	28.90±0.93	1.44	<0.001
Niacin	1.950±0.11	0.967±0.06	0.12	0.001
Pantothenic acid	31.82±0.39	10.18±0.33	0.51	<0.001
Pyridoxine	2.688±0.10	2.082±0.09	0.13	<0.01
α-Tocopherol	205.9±5.79	107.0±3.34	7.88	<0.01

DM, dry matter; SEM, Standard error of means.

**Table 2:** Fermentation characteristics of alfalfa and Chinese leymus silages

Items	Treatment		SEM	Significance1)		
	
	Control	LP		F	T	F× T
pH value						
Alfalfa	5.22^aA^±0.02	4.82^bA^±0.05	0.018	<0.001	<0.001	0.012
Chinese leymus	4.20^aB^±0.04	4.04^bB^±0.00				
Lactic acid (g/kg DM)						
Alfalfa	67.0^bA^±1.64	79.9^aA^±1.40	0.706	<0.001	<0.001	0.030
Chinese leymus	36.3^bB^±0.33	41.3^aB^±0.60				
Acetic acid (g/kg DM)						
Alfalfa	21.7^bA^±0.05	26.2^aA^±0.20	0.478	<0.001	<0.001	0.194
Chinese leymus	6.37^bB^±1.29	13.6^aB^±1.08				
Propionic acid (g/kg DM)						
Alfalfa	11.7^a^±0.36	3.89^bB^±0.34	0.170	0.002	<0.001	<0.001
Chinese leymus	10.8^a^±0.27	8.75^bA^±0.23				
Butyric acid (g/kg DM)						
Alfalfa	0.84^A^±0.06	0.79^A^±0.00	0.012	<0.001	<0.001	<0.001
Chinese leymus	0.55^aB^±0.01	0.11^bB^±0.01				
Lactic acid:acetic acid ratio						
Alfalfa	3.08^B^±0.08	3.05±0.07	0.106	<0.001	<0.001	<0.001
Chinese leymus	7.14^aA^±0.20	3.01^b^±0.43				
Ammonia nitrogen (g/kg TN)						
Alfalfa	165.9^aA^±3.62	120.2^bA^±3.84	1.428	<0.001	<0.001	<0.001
Chinese leymus	23.4^B^±1.87	18.7^B^±0.63				
Flieg’s point						
Alfalfa	68.2^bB^±0.48	83.9^aA^±2.62	0.823	<0.001	<0.001	0.032
Chinese leymus	129.5^bA^±1.14	136.4^aB^±0.30				

LP, *Lactobacillus plantarum* inoculant; SEM, standard error of means; DM, dry matter; TN, total nitrogen.

1) F, forage type; T, treatment; F×T, interaction between forage type and treatment.

Means within the same row (^a,b^) or within the same column (^A,B^) with difference superscripts differ significantly from each other (p<0.05).

**Table 3:** Chemical compositions of alfalfa and Chinese leymus silages

Items	Treatment		SEM	Significance1)		
	
	Control	LP		F	T	F×T
DM (g/kg)						
Alfalfa	359.9^B^±2.35	359.8^B^±5.03	1.169	<0.001	0.787	0.737
Chinese leymus	462.2^A^±1.62	463.7^A^±0.47				
WSC (g/kg DM)						
Alfalfa	7.80^B^±0.35	6.64^B^±0.70	0.500	<0.001	0.078	0.396
Chinese leymus	14.7^A^±1.58	11.73^A^±0.01				
CP (g/kg DM)						
Alfalfa	219.4^bA^±0.23	226.1^aA^±2.71	0.542	<0.001	0.028	<0.001
Chinese leymus	67.1^B^±0.79	66.6^B^±0.34				
NDF (g/kg DM)						
Alfalfa	364.0^B^±5.93	354.5^B^±8.38	3.673	<0.001	0.143	0.749
Chinese leymus	604.5^A^±5.43	590.1^A^±9.00				
ADF (g/kg DM)						
Alfalfa	279.1^A^±3.53	269.1^B^±5.96	1.975	<0.001	0.048	0.901
Chinese leymus	316.3^aA^±0.57	307.4^bA^±0.70				
HC (g/kg DM)						
Alfalfa	84.90^B^±3.80	85.35^B^±2.98	1.970	<0.001	0.849	0.777
Chinese leymus	288.1^A^±5.09	285.8^A^±6.18				

LP, *Lactobacillus plantarum* inoculant; SEM, standard error of means; DM, dry matter; WSC, water-soluble carbohydrate; CP, crude protein; NDF, neutral detergent fiber; ADF, acid detergent fiber; HC, hemicellulose.

1) F, forage type; T, treatment; F×T, interaction between forage type and treatment.

Means within the same row (^a,b^) or within the same column (^A,B^) with difference superscripts differ significantly from each other (p<0.05).

**Table 4:** Vitamin concentrations of alfalfa and Chinese leymus silages

Items	Treatment		SEM	Significance1)		
	
	Control	LP		F	T	F×T
Thiamin (mg/kg DM)						
Alfalfa	2.475^a^±0.04	1.663^bB^±0.09	0.044	0.012	<0.001	0.006
Chinese leymus	2.438^a^±0.08	2.158^bA^±0.06				
Riboflavin (mg/kg DM)						
Alfalfa	52.21^aA^±0.55	42.53^bA^±0.15	0.162	<0.001	<0.001	<0.001
Chinese leymus	11.50^aB^±0.44	10.09^bB^±0.09				
Niacin (mg/kg DM)						
Alfalfa	0.743^aA^±0.03	0.558^bA^±0.03	0.017	<0.001	0.001	0.396
Chinese leymus	0.340^aB^±0.05	0.093^bB^±0.02				
Pantothenic acid (mg/kg DM)						
Alfalfa	18.38^aA^±0.40	14.89^bA^±0.07	0.109	<0.001	<0.001	<0.001
Chinese leymus	4.043^aB^±0.15	3.425^bB^±0.04				
Pyridoxine (mg/kg DM)						
Alfalfa	1.140^A^±0.12	1.108^A^±0.06	0.027	<0.001	0.952	0.613
Chinese leymus	0.700^B^±0.02	0.725^B^±0.02				
α-Tocopherol (mg/kg DM)						
Alfalfa	142.6^b^±6.96	189.4^a^±8.94	3.926	0.591	0.030	0.020
Chinese leymus	162.8±7.38	160.4±2.02				

LP, *Lactobacillus plantarum* inoculant; SEM, standard error of means; DM, dry matter.

1) F, forage type; T, treatment; F×T, interaction between forage type and treatment.

Means within the same row (^a–c^) or within the same column (^A–B^) with difference superscripts differ significantly from each other (p<0.05).
